# Effect of corticosteroids on the clinical course of community-acquired pneumonia: a randomized controlled trial

**DOI:** 10.1186/cc10103

**Published:** 2011-03-15

**Authors:** Silvia Fernández-Serrano, Jordi Dorca, Carolina Garcia-Vidal, Núria Fernández-Sabé, Jordi Carratalà, Ana Fernández-Agüera, Mercè Corominas, Susana Padrones, Francesc Gudiol, Frederic Manresa

**Affiliations:** 1 Respiratory Medicine Department, Hospital Universitari de Bellvitge, Institut d'Investigació Biomèdica de Bellvitge (IDIBELL), University of Barcelona, Feixa Llarga s/n, L'Hospitalet de Llobregat 08907, Barcelona, Spain; 2 CIBER de Enfermedades Respiratorias ISCIII, Madrid, Spain (Spanish Network for the Research in Respiratory Diseases), Recinto Hospitalario Joan March, Carretera Sóller Km 12; 07110 Bunyola, Mallorca, Spain; 3 Infectious Disease Department, Hospital Universitari de Bellvitge, Institut d'Investigació Biomèdica de Bellvitge (IDIBELL), University of Barcelona, Feixa Llarga s/n, L'Hospitalet de Llobregat 08907, Barcelona, Spain; 4 REIPI (Spanish Network for the Research in Infectious Diseases), Fundación Reina Mercedes, Edificio de los laboratorios 6a pl; Av. Manuel Siurot s/n; 41013 Sevilla, Spain; 5 Immunology Department, Hospital Universitari de Bellvitge, Institut d'Investigació Biomèdica de Bellvitge (IDIBELL), University of Barcelona, Feixa Llarga s/n, L'Hospitalet de Llobregat 08907, Barcelona, Spain

## Abstract

**Introduction:**

The benefit of corticosteroids as adjunctive treatment in patients with severe community-acquired pneumonia (CAP) requiring hospital admission remains unclear. This study aimed to evaluate the impact of corticosteroid treatment on outcomes in patients with CAP.

**Methods:**

This was a prospective, double-blind and randomized study. All patients received treatment with ceftriaxone plus levofloxacin and methyl-prednisolone (MPDN) administered randomly and blindly as an initial bolus, followed by a tapering regimen, or placebo.

**Results:**

Of the 56 patients included in the study, 28 (50%) were treated with concomitant corticosteroids. Patients included in the MPDN group show a more favourable evolution of the pO2/FiO2 ratio and faster decrease of fever, as well as greater radiological improvement at seven days. The time to resolution of morbidity was also significantly shorter in this group. Six patients met the criteria for mechanical ventilation (MV): five in the placebo group (22.7%) and one in the MPDN group (4.3%). The duration of MV was 13 days (interquartile range 7 to 26 days) for the placebo group and three days for the only case in the MPDN group. The differences did not reach statistical significance. Interleukin (IL)-6 and C-reactive protein (CRP) showed a significantly quicker decrease after 24 h of treatment among patients treated with MPDN. No differences in mortality were found among groups.

**Conclusions:**

MPDN treatment, in combination with antibiotics, improves respiratory failure and accelerates the timing of clinical resolution of severe CAP needing hospital admission.

**Trial Registration:**

International Standard Randomized Controlled Trials Register, ISRCTN22426306.

## Introduction

Despite advances in diagnostic methods and antibiotic treatment, community-acquired pneumonia (CAP) remains an important cause of mortality [1-3]. In the industrialized countries, CAP is the sixth highest cause of mortality and the first among infectious diseases. Although mortality in patients with CAP fell dramatically with the introduction of antibiotics in the 1950s, since then it has remained relatively stable. Current series report an overall mortality rate of 8 to 15% [4-6].

A recent study [7] of the factors associated with early death in patients with CAP reinforces the classical concept that some deaths were not due to failure to eradicate the microorganism causing CAP, but are closely related to inadequate host response [8]. Excessive cytokine response in patients with severe CAP has been linked in many previous studies with deleterious effects and poor prognosis [9-13].

In this context, the use of immunomodulation appears to be an appealing option for improving prognosis in CAP. Theoretically, an anti-inflammatory treatment given prior to antibiotic therapy could prevent an excessive inflammatory response, improving the prognosis of more severe episodes of CAP. Therefore, the use of corticosteroids as an adjunct therapy for pneumonia has been a matter of debate [14-16]. Corticosteroids are known to reduce the production of the main inflammatory cytokines (TNFα, IL-1β, IL-8, and IL-6), and the subsequent recruitment of inflammatory cells into the alveolar space leading to a more equilibrated response.

Here we conducted a prospective, randomized, double blind, placebo-controlled trial to analyse whether a corticosteroid therapy, administered in the form of a methyl-prednisolone bolus given prior to antibiotic treatment followed by sustained infusion for nine days, was able to modulate the inflammatory response and clinical outcome of selected hospital-admitted CAP patients presenting respiratory failure and extensive radiological consolidations.

## Materials and methods

### Setting, study design and subjects

This study was conducted at the Hospital Universitari de Bellvitge, a 900-bed hospital in Barcelona, Spain, which serves a population of about 1,100,000 people. The study was prospective, double-blind and randomized. Patients admitted to the hospital with CAP, and who met the selection criteria and agreed to participate in the study, were assigned to receive either placebo or methyl-prednisolone (MPDN) in combination with empirical antibiotic treatment.

CAP was diagnosed according to conventional criteria previously reported elsewhere [9]. Inclusion criteria were: 1) extensive radiological consolidations (completely affecting at least two lobes); and 2) respiratory failure (pO2/FiO2 <300). Exclusion criteria included: 1) age <18 years and >75 years; 2) no written informed consent available; 3) known hypersensitivity to steroids; 4) steroid treatment in the previous 48 h; 5) need for steroid treatment for any reason (asthma, chronic obstructive pulmonary disease (COPD), and so on); 6) uncontrolled diabetes mellitus; 7) active peptic ulcer; 8) active mycobacterial or fungal infection; 9) reported severe immunosuppression; 10) hospital admission during the previous eight days; 11) empyema; 12) extrapulmonary septic manifestations; 13) presence of shock; 14) pre-mortem status; 15) aspiration pneumonia; and 16) need for mechanical ventilation (MV) prior to inclusion in the study.

The study was carried out in accordance with the Helsinki Declaration of 1975, as revised in 1983. Written informed consent was obtained in all cases from patients or their relatives. The study was approved by the Review Board Committee of our institution and by the Agencia Española del Medicamento (trial identification number AEM99/0145). The trial has also been inscribed in the International Standard Randomized Controlled Trials Register (ISRCTN22426306).

### Interventions

We aimed to analyze the effect of a steroid treatment on the clinical course and outcome of CAP needing hospital admission, as well as on the profile of the host inflammatory response. For this propose we conducted a randomized, double blind, controlled trial. Patients who were placed on systemic steroid therapy were compared with those who received a placebo at the time of diagnosis. All patients received intravenous antibiotic treatment consisting of 1 g/day of ceftriaxone and 500 mg/day of levofloxacin. In addition, a bolus of 200 mg of MPDN or placebo was administered, 30 minutes before starting the antibiotic treatment. Thereafter, a maintenance intravenous dose (20 mg/6 h) was given for three days, then 20 mg/12 h for three days, and finally 20 mg/day for another three days. The placebo formulation was kindly provided by Sanofi-Aventis (Paris, France) and had a physical appearance similar to the corticosteroid drug. Omeprazole was administered to patients to minimize the side effects of steroids and, if necessary, insulin therapy was started to control blood glucose levels. Intravenous ceftriaxone was maintained for nine days. After five days, intravenous levofloxacin was sequentially switched to 500 mg by oral route for at least 20 days.

The main clinical variables were monitored during the first nine days of admission. The clinical course was assessed by the time to resolution of morbidity (TRM) score, a semi-quantitative score that combines clinical and radiological variables in order to determine the timing of improvement after inclusion [14]. In addition, chest X-ray, and routine venous blood tests (cell counting, biochemistry, C-reactive protein (CRP), and arterial blood gases analyses were obtained on days 1, 2, 3, 5 and 7 after entry. All patients were monitored one month after discharge. Radiological analysis and clinical follow-up were carried out by independent clinicians. The parameters used to calculate the TRM score, as well as the methodology for its application are described elsewhere [17].

The presence of respiratory failure requiring conventional MV or non-invasive positive pressure ventilation (NPPV) was selected as the primary outcome of the study. The secondary endpoint of this study was to assess the evidence of benefit in terms of an improved clinical course measured by pO2/FiO2 ratio, radiological improvement, TRM score, length of hospital stay, length of ICU stay, mortality and decreasing levels of systemic inflammatory response (IL-6, TNF-α, IL-8, IL-10 and CRP).

### Microbiological studies

The investigation of pathogens in blood, normally sterile fluids, sputum, and other samples was performed by standard microbiological procedures. The *Streptococcus pneumoniae *antigen in urine was detected by using a rapid immunochromatographic assay (Now™, Binax, Inc., Portland, ME, USA). *Legionella pneumophila *serogroup I antigen in urine was detected using an immunochromatographic method (NOW Legionella Urinary Antigen Test; Binax Inc.) or enzyme-linked immunosorbent assay (ELISA-Bartels, Bartels, Trinity Biotech, Wicklow, Ireland). Standard serologic methods were used to determine antibodies against atypical agents. The criteria for classification of pneumonia (for example: definitive, probable) have been described elsewhere [18].

### Study of the inflammatory response

In all cases, serial venous blood samples were obtained at entry, before initial treatment, and on days 1, 2, 3, 5 and 7 after inclusion. Circulating pro-inflammatory (TNF-α, IL-6, IL-8) and anti-inflammatory (IL-10) cytokines were determined according to previously described methodology [9].

### Sample size calculation

By using a two-tailed test and assuming a 90% follow-up, it was calculated that 56 episodes would be needed (28 in each group) to detect a difference of 15% in the need of mechanical ventilation between the control group and intervention group, the one treated with corticosteroids (80% power, 5% significance level).

### Statistical analysis

The results of the comparative analysis of serial measurements (clinical variables, cytokine levels) and different scores (simplified acute physiology score (SAPS), radiological and clinical) at entry and after successive days on MPDN or placebo are expressed as median, interquartile range, first and third quartile. Significance levels were set at 0.05. Baseline data between the two therapeutic groups were compared by means of the non-parametric Mann-Whitney U test for continuous data, and by the Cochran-Mantel-Hansel chi square test for categorical data. The chi-square test and Kruskal-Wallis non-parametric tests were used to compare response groups. For 2 × 2 tables where any cell contained fewer than five observations, Fisher's exact two-tailed test for categorical data was used. Data for the primary and secondary end-points were analysed on intention-to-treat-analysis.

All statistical calculations were performed using the Statistical Package for the Social Sciences (Version SPSS 15.01s) for Windows (SPSS Inc, Chicago, IL. USA).

## Results

**Figure 1:**
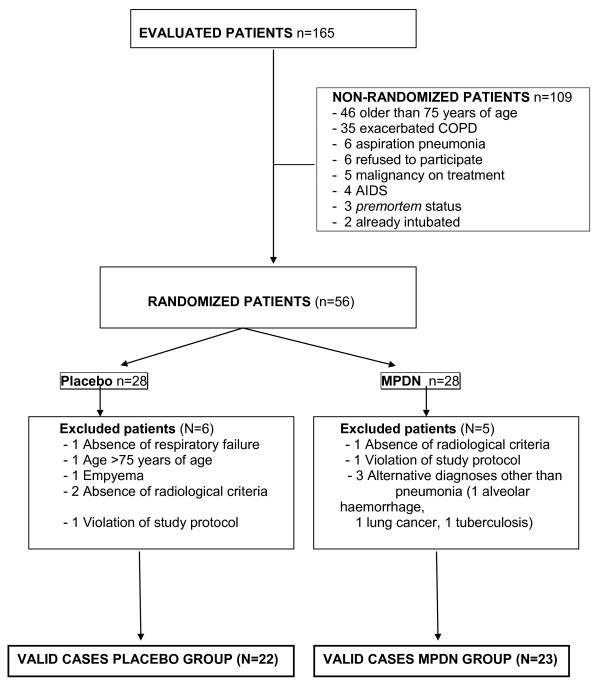
**Selection of patients for the study**.

Over a three-year period, 165 consecutive patients presenting with CAP and admitted to our institution were considered for inclusion into the study (Figure 1). After evaluation, a total of 56 episodes were randomly assigned and included in an intention-to-treat-analysis. The baseline clinical and radiological characteristics of these cases are summarized in Table 1.

**Table 1:** Characteristics of valid cases (*n *= 45)

	Placebo	MPDN	*P*
**N patients**	22 (14 m/8 f)	23 (16 m/4 f)	ns
**Age (years)**	61 (48 to 66)	66 (49 to 70)	ns
**Comorbidity conditions**			
COPD	2	4	ns
Cardiovascular disease	2	4	ns
Diabetes melllitus	4	2	ns
**Symptoms**			
Fever >38.5°C	18	20	
Cough	14	18	ns
Breathlessness	17	16	ns
Expectoration	10	10	ns
Chest pain	10	11	ns
Chills	13	14	ns
Altered mental status	0	0	ns
Duration of symptoms (days)	5 (3 to 8)	5 (3 to 7)	ns
**Clinical signs**			
Temperature*	38.6 (38 to 39)	38.5 (37.6 to 39.5)	ns
Heart rate*	102 (96 to 125)	109 (100 to 120)	ns
Respiratory rate*	32 (30 to 40)	35 (30 to 38)	ns
**Blood tests**			
White cell × 10^9^*	10.2 (7.4 to 13.5)	13.5 (11.4 to 15.6)	0.01
Urea (mmol/dl) *	7 (5 to 12)	9 (7 to 12)	ns
pO2/FiO2*	257 (209 to 276)	200 (233 to 236)	ns
**Radiological findings**			
Bilobar	11 (50%)	15 (65%)	ns
Multilobar	11 (50%)	8 (35%)	ns
**Previous antibiotic treatment**	5 (23%)	4 (17%)	ns
SAPS*	7 (6 to 12)	8 (5 to12)	ns
**Fine Score**			
I	0 (0%)	0 (0%)	ns
II	3 (14%)	1 (4%)	ns
III	7 (32%)	6 (26%)	ns
IV	11 (50%)	14 (61%)	ns
V	1 (4%)	1 (4%)	ns

*median and interquartile range, ns: no statistical significance (*P *>0.05)

MPDN: methyl-prednisolone. SAPS: Simplified acute physiology score.

Data concerning the microbiological findings are summarized in Table 2. *Streptococcus pneumoniae *and *Legionella pneumophila *were the most common aetiologies. No statistically significant differences in aetiology were observed between the two groups, although pneumococcal pneumonia was more frequent in the placebo group. A definitive etiological diagnosis was obtained in 25 (55.6%) cases and a presumptive diagnosis in 11 (24.4%) additional episodes. No etiological diagnosis could be made in nine (20%) cases.

**Table 2:** Causative organisms

Microorganisms	Placebo	MPDN	Total	*P*
***Streptococcus pneumoniae***	10 (45%)	5 (22%)	**15**	ns
Sputum	1			
Sputum + urinary antigen	1	1		
Urinary antigen	3	3		
Blood culture	1	1		
Sputum + blood culture+ urinary antigen	2			
Blood culture + urinary antigen	2			
***Legionella pneumophila***	5 (23%)	7 (30%)	**12**	ns
Sputum + urinary antigen	1			
Urinary antigen		3		
Sputum + serology	1			
Sputum + urinary antigen + serology		1		
Urinary antigen + serology		3		
Serology	3			
***Haemophilus influenzae***				
Sputum	1 (4%)	1 (4%)	**2**	ns
***Streptococcus viridans***				
Blood culture		1 (4%)	**1**	ns
**Atypical pathogens ***(serology)*			**2**	ns
**Mycoplasma pneumoniae**	1 (4%)	1 (4%)	**2**	ns
***- Chlamydia pneumoniae***		2 (9%)	**2**	ns
***- Chlamydia psittaci***	1 (4%)		**1**	ns
***- Coxiella burnetti***		1 (4%)	**1**	ns
**No etiological diagnosis**	4 (18%)	5 (22%)	**9**	ns

MPDN, methyl-prednisolone; (*) ns, no statistical significance.

**Figure 2:**
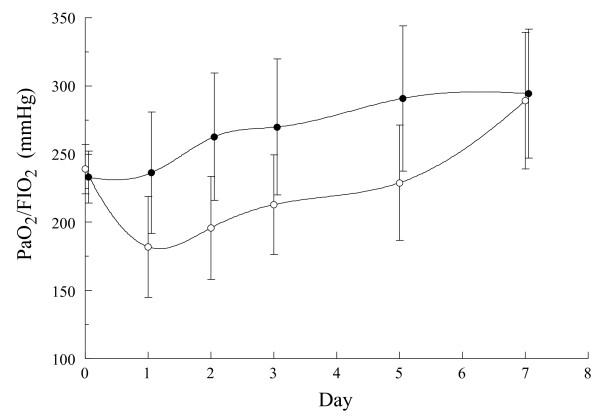
**Comparative evolution of paO_2_/FIO_2 _ratio over the days of treatment and between the two study groups**. Mean values with 96% Confidence Intervals. Open circles: Placebo. Closed circles: methyl-prednisolone (MPDN). Line: Clamp Spline Interpolation. (*P *= 0.001 Kruskal-Wallis one-way non-parametric test).

The outcomes of patients are shown in Table 3. Patients included in the MPDN group show a more favourable evolution of the pO2/FiO2 ratio (Figure 2), faster decrease of fever, as well as higher radiological improvement at seven days (*P *< 0.05). The TRM was also significantly shorter in this group: median 5 days (interquartile range (IQR) 2 to 6) vs. 7 days (IQR 3 to 10), respectively. Six patients met the criteria for MV: five in the placebo group (22.7%) and one in the MPDN group (4.3%). NPPV was initially attempted in all these cases, but only proved successful in three (two in the placebo group and one in the MPDN group). Conventional MV was eventually required in three cases, all of them belonging to the placebo group. The duration of MV was 13 days (IQR 7 to 26 days) for the placebo group and 3 days for the only case in the MPDN group. The differences do not reach statistical significance. In the intention-to-treat analysis the comparison of all these variables in the two groups obtained similar results.

**Table 3:** Main outcome variables

	Placebo	MPDN	*P*
**Need for mechanical ventilation**			
Conventional mechanical ventilation (‡)	3	0	ns
Non-invasive positive pressure ventilation (‡)	2	1	ns
Mechanical ventilation total (‡)	5	1	ns
Duration of mechanical ventilation (days):			
- Conventional * (†)	10 (13 to 19.5)	-	ns
- NPPV * (†)	16.5 (6 to 27)	3	ns
- Total * (†)	13 (7 to 26)	3	ns
**ICU admission**	5	4	ns
Duration of ICU stay, days (†)	10.5 (6.25 to 24.5)	6.5 (5.5 to 9)	ns
<24 hours (‡)	3	3	ns
>24 hours (‡)	2	1	ns
Development of shock (‡)	2	1	ns
**Mortality**			
Early ( ≤ 9 days) (‡)	-	1	ns
Late (>9 days) (‡)	1	0	ns
**General ward stay **(†)	11.5 (9 to 14)	10 (9 to 13)	ns
**Total hospital stay **(†)	12 (9 to 18)	10 (9 to 13)	ns
**Time to resolution of morbidity **(†)	7 (3 to 10)	5 (2 to 6)	0.02

*median and interquartile range; ns:no statistical significance (p > 0.05).

(†) non paparametric Mann-Whitney U test.

(‡) Fisher exact two-tailed test *median and interquartile range.

ICU, intensive care unit; MPDN, methyl-prednisolone; NPPV, non-invasive positive pressure ventilation.

Three patients in each study group were admitted to the ICU within the first 24 h after hospital admission. Subsequently, another two patients from the placebo group and one in the MPDN group were transferred to ICU. Of these nine patients, three developed septic shock, two of them were from the placebo group. The duration of ICU stay tended to be longer in the placebo group compared to the MPDN group: 10.5 *vs. *6.5 days. There were no significant differences in the general ward stay and the total length of hospital stay. No differences in mortality were found among groups.

**Figure 3:**
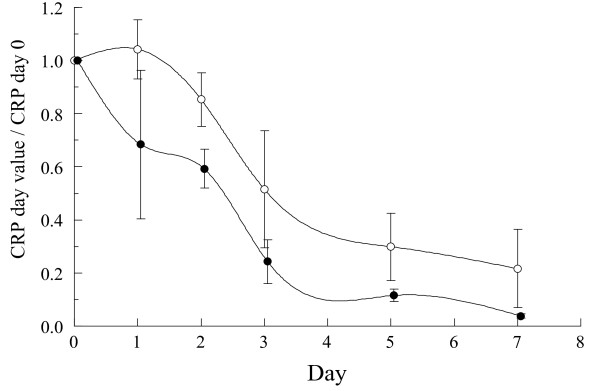
**Comparative evolution of C-reactive protein ratio over the days of treatment and between the two study groups**. The CPR ratio was calculated by dividing every day value by the CPR value at Day 0. Mean values with 96% confidence Intervals. Open circles: Placebo. Closed Circles: methyl-prednisolone (MPDN). Line: Clamp Spline Interpolation. (*P* = 0.05 Kruskal-Wallis one-way non-parametric test).

In relation to the intensity of the inflammatory response, when comparing the evolution of cytokine levels between the two groups, IL-6 showed a significantly quicker decrease after 24 h of treatment among patients treated with MPDN (Table 4). In addition (Figure 3), the CRP ratio displayed a similar trend, reaching statistical significance (*P *= 0.04, Kruskall-Wallis one-way non-parametric test).

**Table 4:** Plasma cytokine concentrations (pg/ml)*

	Admission	Day 1	Day 2	Day 3	Day 5	Day 7	***P *(**†)
**IL-6**							*P *= 0.0001
Placebo	489.7 (83.5 to 2700)	219 (54 to 691)	77.5 (35.9 to 266.7)	48 (17.5 to 136)	37 (15.2 to 104.2)	23.9 (10.2 to 77.4)	
MPDN	1060 (143.7 to 2594)	40.6 (20.8 to 132)	12.2 (0 to 36.4)	11.3 (0 to 34)	9 (0 to 23)	0.5 (0 to 23)	
**IL-8**							*P *= 0.01
Placebo	118 (28.1 to 253)	48.6 (19.9 to 196)	38.8 (16.1 to 92)	20.3 (12 to 103)	22.5 (9.5 to 66.9)	14 (0 to 55.2)	
MPDN	134 (68.2 to 226)	32.3 (19.5 to 75)	13.7 (7.4 to 35)	14.6 (5.8 to 24)	11.3 (6.2 to 23.8)	11 (0 to 53)	
**IL-10**							ns
Placebo	9.9 (0 to 62.2)	0 (0 to 11.2)	0 (0 to 4)	0 (0 to 5)	0 (0 to 3.7)	0 (0 to 2.7)	
MPDN	14.8 (0 to 35.2)	0 (0 to 6)	0 (0 to 5)	0 (0 to 0)	0 (0 to 0)	0 (0 to 0)	

* Median (pg/ml), interquartile range (first and third quartile).

(†) Kruskall-Wallis one-way non-parametric test (*P *< 0.05).

IL-6, interleukin-6; IL-8, interleufin-8; IL-10, interleukin-10; MPDN, methyl-prednisolone.

Complications related to the steroid treatment were minimal: among the 23 patients of the MPDN group, only one needed insulin for adequate diabetes control. Additionally, one patient suffered a digestive haemorrhage related to an active peptic ulcer 12 days after inclusion in the study (3 days after MPDN and omeprazole had been discontinued). The patient did well following a conservative approach.

## Discussion

Few data have been published about the use of corticosteroids as an adjuvant anti-inflammatory treatment in CAP [14-16,19]. In order to demonstrate the hypothetical benefit of this strategy, we designed this prospective, double-blind, randomized study of patients with CAP and admitted because of: 1) large pulmonary consolidation; and 2) acute respiratory failure. Our results indicate that the administration of an adjuvant steroid therapy in combination with ceftriaxone plus levofloxacin significantly improved several clinical course variables such as the pO2/FiO2 ratio, the degree of radiological resolution and TRM score. In addition, some inflammatory markers such as IL-6 and CRP showed significantly lower blood concentrations and a more favourable time-course in the MPDN group. Mechanical ventilation was needed in only one episode from the MPDN group compared with five cases in the control group, while the duration of ICU stay showed a clear trend in favour of the MPDN group. However, these differences did not reach statistical significance.

The need for MV was chosen as the major endpoint for this trial and was preferred over mortality, as it appears to be a more multi-factor variable than the development of severe respiratory failure. Sample size calculation was determined on the basis of the findings reported by a limited number of studies [20,21] and our own clinical experience. It would appear that the sample size is too small to confer statistical significance to the observed differences in this endpoint, but, were these differences to be maintained, a 50% larger sample size could be enough to achieve statistical significance. Nevertheless, the number of studied cases was enough to demonstrate significant differences in other relevant clinical variables, in particular the pO2/FiO2 ratio.

Some studies have previously evaluated the impact of corticosteroid treatment in the prognosis of patients with CAP. In 1993, Marik *et al. *[22] postulated that a low dose of hydrocortisone given prior to antibiotic therapy in ICU-admitted CAP patients could prevent the second wave of TNF-a release in the blood; however, the authors were unable to confirm this hypothesis and concluded that the hydrocortisone treatment had no effect on the serum TNF-a levels or on the clinical course of patients. In another study, Monton *et al. *[23] reported that a prolonged steroid treatment decreased systemic and lung inflammatory responses in patients with severe pneumonia, with a tendency to decrease mortality. Confalonieri *et al. *[15] evaluated the effect of steroids on ICU-admitted CAP patients with respiratory failure or shock; they conducted a randomized, double-blind placebo-controlled trial and concluded that a seven-day course of low-dose hydrocortisone infusion was associated with a significant reduction in the duration of MV, length of hospital stay and hospital mortality. The inclusion criteria of patients, with more severe disease (all patients with ICU admission and 74% requiring mechanical ventilation) differed markedly from the current cohort. In this setting, Salluh *et al.*[24]reported that most patients with severe CAP admitted to the ICU had adrenal insufficiency caused by a disregulation of the hypothalamic-pituitaryadrenal axis. Clearly, the presence of underlying adrenal insufficiency could explain the favourable results obtained among some of the patients with severe pneumonia. Our study, carried out in a less severe form of CAP also confirms a beneficial effect for corticosteroids in association with the antibiotic treatment. In another series, Garcia-Vidal *et al. *[19] also documented, in a retrospective observational analysis of 308 patients with CAP, that treatment with systemic steroids decreased mortality in the patients with severe CAP who received simultaneous administration of steroids. Very recently, another randomized and double-blinded study [16] comparing the efficacy of 40 mg of prednisone, in combination with the antibiotic treatment, given during seven days in a series of 213 patients presenting CAP of different levels of severity, concluded that the corticoid treatment did not improve the outcome of the episodes. Nevertheless, in this study the percentage of severe episodes was lower than ours, the administered antibiotic regimen was not homogeneous, and the number of *Legionella *episodes was very low, with only one case receiving prednisone. At the end, these authors concluded that a benefit of corticosteroids in the more severe episodes cannot be excluded.

The dosage and duration of corticosteroid treatment is a matter for debate. In our study we decided to administer an initial bolus of MPDN followed by tapering for nine days; this is a similar schedule to that used in daily clinical practice when treating exacerbated COPD. In other series [22,23], hydrocortisone was preferred, but at variable dosages. The dosage and timing of administration is probably more important than the characteristics of the chosen molecule. We incorporated the strategy of prescribing an initial MPDN bolus 30 minutes before the first dose of the antibiotic combination in order to interfere with the pro-inflammatory wave induced by sudden bacterial killing. Although it is possible that a lower dosage of corticosteroids could obtain a similar effect, we believe that the use of a higher dose may be justified until a favourable effect has been demonstrated.

The effects of steroids on the immune system are many and complex. Corticosteroids are known to reduce the production of the main inflammatory cytokines (TNFα, IL-1β, IL-8, and IL-6), and the subsequent recruitment of inflammatory cells into the alveolar space leading to a more equilibrated response. Glucocorticoids inhibit cytokines and other inflammatory molecules stimulated by bacterial infections that could be harmful to the host. However, the use of steroids also exerts a decisive influence in the immune function of macrophages and granulocytes, the main cell host defences against bacteria [25-27]. In this context, it seems clear that advances in the knowledge of cytokines release and kinetics, gamma interferon and G-CSF, will permit a better understanding of the interaction between the endocrine and immune systems in respiratory infection and will make it possible to identify the subset of patients in whom steroids administration would be safe and effective.

Despite a number of strengths, our prospective, double-blind and randomized study has certain limitations that should be acknowledged. First, the study included a relatively small number of patients. Second, the strict exclusion criteria (such as >75 years, presence of severe immunosuppression, presence of shock, pre-mortem status, aspiration pneumonia or the need for MV prior to inclusion in the study) may explain the low mortality observed in our study. This reason precludes analysing the impact of the use of corticosteroids on mortality in these patients. Third, our conclusions apply only to a subset of patients with CAP and extensive radiological consolidations and/or respiratory failure. It should be noted that there were several significant exclusion criteria, such as the need for steroid use for any reason (asthma, COPD, and so on), shock, and the need for MV prior to inclusion in the study among others. Finally, the administration of systemic steroids occurred at different times in the course of the disease. Timing of steroid administration might play a critical role because inflammatory response is a dynamic process and excessive modulation of any pathway could be the cause of an unwanted response.

## Conclusions

The results provided by this double-blind, randomised trial of CAP patients admitted to a general hospital ward and presenting severe respiratory failure and extensive radiological consolidations support the hypothesis that an adjuvant steroid therapy decreases the inflammatory response, and seems to reduce the need for MV. This experience supports the need for larger studies in order to establish the usefulness of this therapeutic strategy in the different kinds of CAP.

## Key messages

• In this prospective, double-blinded, randomized study comparing methylprednisolone (MPDN) to a placebo combined with ceftriaxone plus levofloxacin in severe CAP, MPDN administration was associated with improved oxygenation, faster decrease of fever and radiological improvement.

• MPDN administration was also associated with a faster reduction in blood IL-6 and CRP levels in the first 24 hours of treatment.

## Abbreviations

CAP: community-acquired pneumonia; COPD: chronic obstructive pulmonary disease; CRP: C-reactive protein; ICU: intensive care unit; ELISA: enzyme linked immunosorbent assay; FiO2: fraction of inspired oxygen; G-CSF: granulocyte colony-stimulating factor; IL-6: interleukin-6; IL-8: interleukin-8; IL-10: interleukin-10; IQR: interquartile range; MPDN: methyl-prednisolone; MV: mechanical ventilation; NPPN: non-invasive positive pressure ventilation; PaO2: partial pressure of oxygen; SAPS: simplified acute physiology score; TNF-α: tumor necrosis factor-α; TRM: time to resolution of morbidity.

## Competing interests

The authors declare that they have no competing interests.

## Authors' contributions

JD contributed to study concept and design. SF, JD, NF, AF and SP contributed to acquisition of data. SF, JD, CG, JC, FG and FM contributed to analysis and interpretation of data. JD, CG, JC, FG and FM contributed to drafting of the manuscript. SF, JD, CG, NF, JC, AF, MC, SP, FG and FM contributed to critical revision of the manuscript for important intellectual content. SF, NF and CG contributed to statistical analysis. JD obtained funding. SF, CG, AF, MC and SP contributed to administrative, technical, and material support. JD, FM, SF, JC, CG and FG contributed to study supervision.
